# Effective management of femoral artery infection using lateral femoral bypass

**DOI:** 10.1016/j.jvscit.2025.101771

**Published:** 2025-03-04

**Authors:** Kotaro Mukasa, Yasunori Yakita, Ryosuke Marushima, Musashi Tsuda, Shinichiro Abe, Soichi Asano

**Affiliations:** Department of Cardiovascular Surgery, Chiba Cardiovascular Center, Ichihara, Chiba, Japan

**Keywords:** Extra-anatomic bypass, Femoral artery pseudoaneurysm, Groin infection, Radiation therapy

## Abstract

Infections of the groin present significant challenges for both infection control and limb salvage. This case report describes a patient with a history of penile cancer treated with radiation therapy, who developed a femoral artery infection following thromboendarterectomy. A lateral femoral bypass was performed, combined with thorough debridement and reconstruction using a rectus abdominis myocutaneous flap. This approach was effective for wound management, infection control, and revascularization. Although this procedure is rarely performed, it can serve as a valuable option for limb salvage in complex cases involving infection and previous radiation exposure.

Groin infection can be life-threatening and may significantly challenge limb salvage. Extra-anatomic bypass surgery offers an effective solution. This case report presents a patient who was successfully treated with a lateral femoral bypass, achieving infection control and effective revascularization. Patient data were reviewed from our electronic database. The patient provided written consent for publishing the case, including images.

## Case report

The patient was a 77-year-old male with a history of radiation therapy for penile cancer 30 years earlier and Dacron Y-grafting for an abdominal aortic aneurysm 8 years previously, with prosthetic replacement extending from the infrarenal aorta to both common iliac arteries. He had been smoking until he came to our outpatient clinic. He presented with claudication and difficulty walking even 10 meters. Contrast-enhanced computed tomography (CECT) revealed occlusion from the right external iliac artery (EIA) to the common femoral artery. Thromboendarterectomy, common femoral artery angioplasty using a bovine pericardial patch, and endovascular treatment of the EIA with a self-expanding nitinol stent were performed.

**Fig 1:**
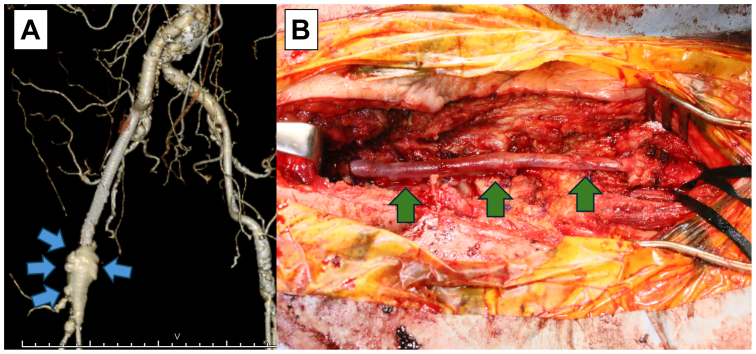
**(A)** Reconstructed three-dimensional computed tomography angiogram showing pseudoaneurysm at the suture line between the patch and artery (*blue arrows*). **(B)** Intraoperative photography showing the revascularization achieved by the bypass from the right external iliac artery (EIA) to the superficial femoral artery (SFA) using a great saphenous vein graft (SVG) (*green arrows*).

Postoperatively, due to the effects of previous radiation therapy, delayed wound healing occurred. Negative pressure wound therapy using standard vacuum-assisted closure was initiated on postoperative day 7 for the management of the dehisced surgical wound. However, optimal wound healing was not achieved. On postoperative day 119, arterial bleeding occurred from the surgical wound. A CECT revealed a pseudoaneurysm, and wound cultures resulted in identification of methicillin-resistant coagulase-negative staphylococci (MRCNS), leading to the diagnosis of a bovine pericardial patch infection, delayed wound healing, and pseudoaneurysm formation (Fig 1, *A*). Broad-spectrum antibiotics (meropenem + teicoplanin) were initiated. Emergency debridement and in situ bypass from the right EIA to the superficial femoral artery (SFA) were performed. The great saphenous vein graft (SVG) was used because it is considered more resistant to infection than synthetic grafts (Fig 1, *B*). The deep femoral artery was ligated as it was perfused through collateral circulation. The newly incised areas at the cranial and caudal sides of the wound, where fresh tissue was exposed, were closed with sutures, but the central area, which had been open preoperatively, was left open.

**Fig 2:**
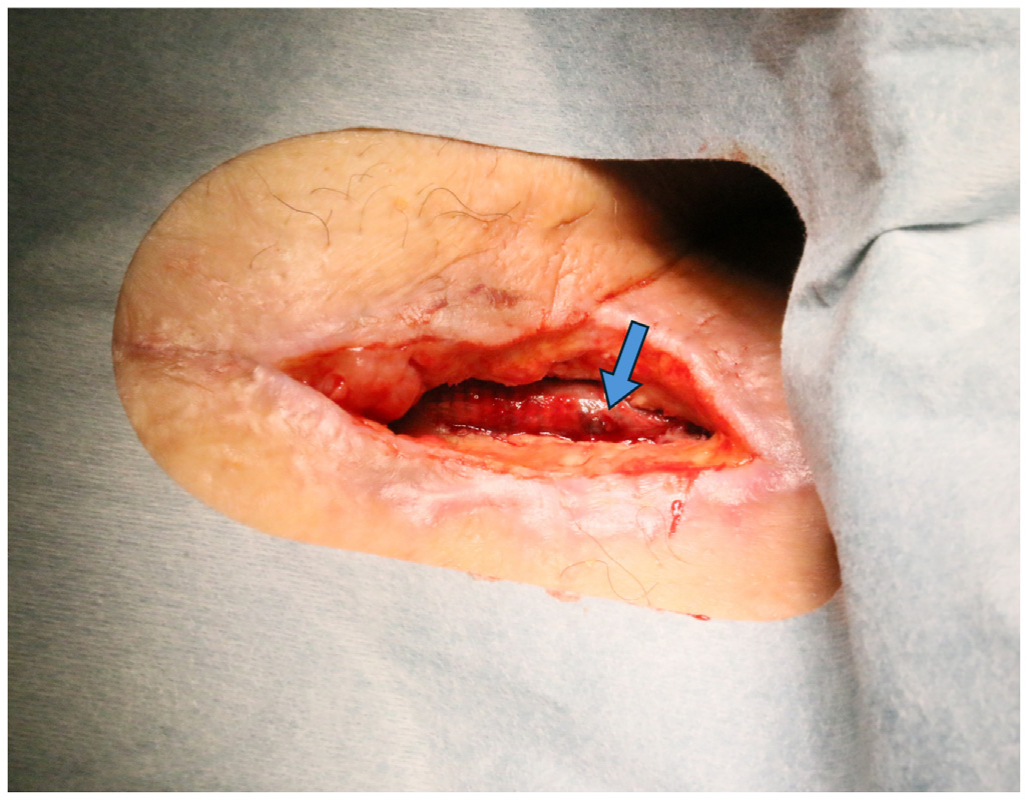
A hole identified in the great saphenous vein graft (SVG) after the placement of the stent graft (*blue arrow*).

Negative pressure wound therapy was resumed, but on postoperative day 23, the patient experienced sudden massive bleeding from the groin wound. Emergency placement of a stent graft was performed to reline the SVG. A hole was found in the midsection of the SVG (Fig 2). Wound cultures again identified MRCNS, suggesting a recurrent infection that had spread to the SVG. Given that bypass reconstruction through the groin was insufficient for infection control, it was decided to perform a bypass from the right limb of the Y-grafting, tunneling laterally around the iliac bone to the mid-thigh SFA, combined with thorough debridement of the groin area.

The procedure was performed under general anesthesia with the patient in a supine position. A 12-cm right paramedian incision was made, approaching the retroperitoneal space laterally to the rectus abdominis muscle. The right limb of the Y-grafting was secured. A 7-cm incision was made in the mid-thigh to expose the SFA between the sartorius and rectus femoris muscles. Two 3-cm incisions were made laterally over the iliac crest, and a tunnel for a 6-mm ringed PROPATEN (W.L. Gore) was created via the lateral thigh. Specifically, the graft route was formed by passing through the rectus abdominis and oblique muscles and crossing over the anterior superior iliac spine. A side-to-end anastomosis was performed between the right limb of the Y-grafting and the PROPATEN graft. A 6 Fr sheath was inserted through the distal SFA wound, and a wire was passed into the right external iliac artery, which was occluded using a 14-mm An Amplatzer Vascular Plug Ⅱ (AVP Ⅱ; Abbott Vascular). The graft was anastomosed to the SFA, and the SFA was ligated proximally to the anastomosis site, cutting off blood flow through the infected groin.

**Fig 3:**
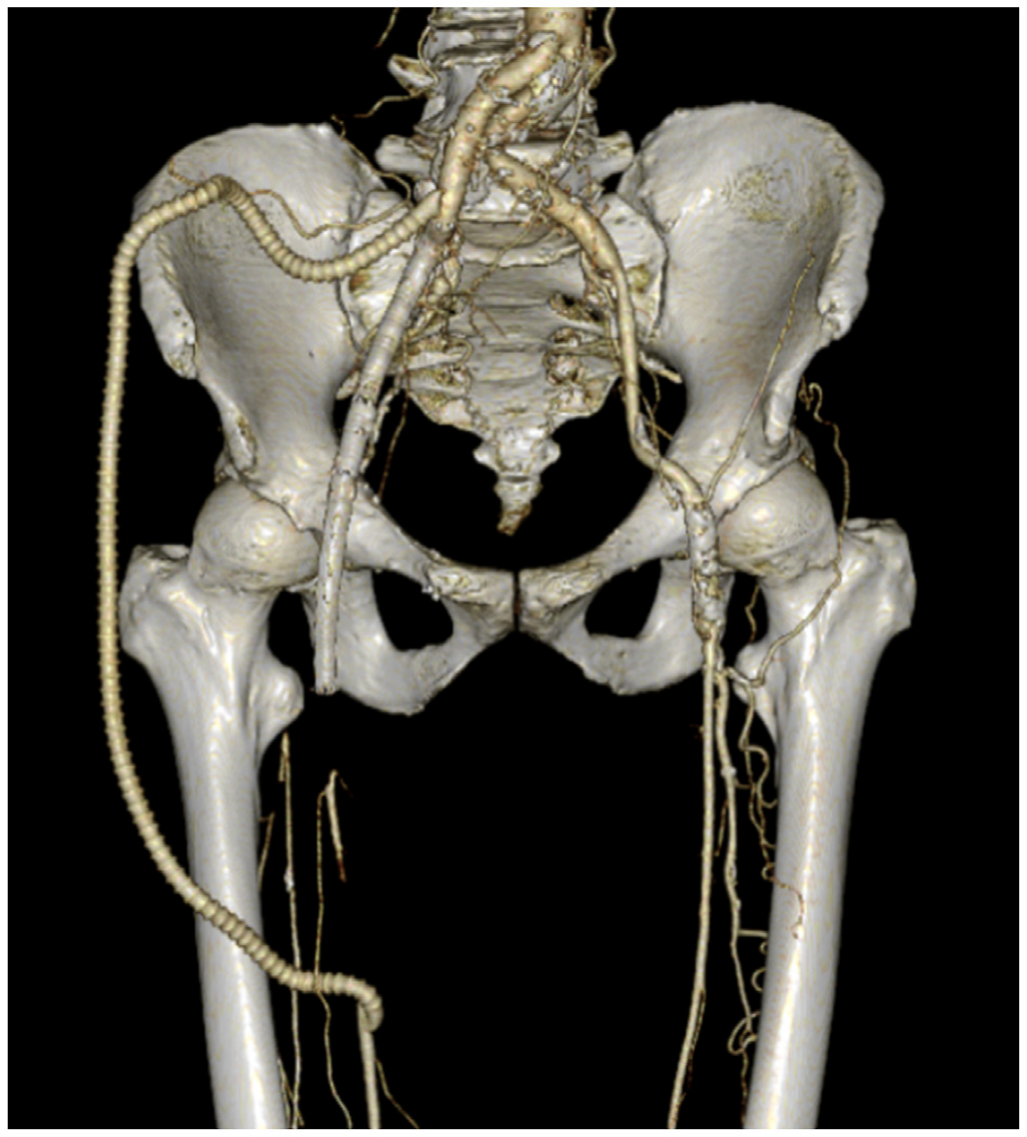
Postoperative reconstructed three-dimensional computed tomography angiogram showing successful revascularization achieved by lateral femoral bypass.

On postoperative day 15, a thorough debridement of the groin was performed, and the SVG and Viabahn stent were removed, taking care to avoid injury to the femoral vein and femoral nerve. A rectus abdominis muscle flap was used for reconstruction. The inferior epigastric artery, along with the surrounding adipose tissue, was preserved as the pedicle, and a rectus abdominis muscle flap measuring approximately 20 cm by 10 cm was harvested and transferred to the groin. The flap healed well, and the patient was discharged after graft patency was confirmed via CECT (Fig 3). Even 6 months after discharge, the patient has remained free of obstructive symptoms while continuing treatment with warfarin and aspirin.

## Discussion

Various techniques for extra-anatomic bypass infection have been reported.^1^ In situations where access to the retroperitoneum is anticipated to be challenging, an axillofemoral-SFA bypass may be considered. However, the longer graft length and increased risk of complications make it a less favorable first-line option.^2^ Additionally, there is a risk of graft compression depending on the patient’s position. This limitation is also seen in lateral femoral bypass surgery. In contrast, an obturator bypass carries a lower risk of graft compression and offers better long-term patency due to its shorter graft length.^1^ However, surgeons must take care to avoid injury to the obturator nerve and artery during the procedure.^3^ In this case, given the patient’s prior radiation therapy and anticipated pelvic adhesions, a lateral femoral bypass was chosen.

Radiation therapy is well-known to delay wound healing.^4^ Furthermore, the groin is a moist environment with a high microbial load, which may have contributed to the infection of the femoral artery and bovine pericardial patch in this case.^5^ MRCNS was isolated from wound cultures. Infection involving the femoral artery can lead to massive and potentially fatal hemorrhage. A more thorough consideration of the possibility of infection was warranted. In cases of previous radiation therapy, as in this case, bypass procedures that avoid irradiated areas or endovascular repair should be considered. Additionally, even for femoral artery angioplasty, the use of autologous vein patches should be considered to reduce the risk of infection.

In situ bypass grafting carries an inherent risk of reinfection. In this case, despite the use of an autologous SVG for in situ bypass, the patient experienced sudden bleeding due to a recurrent infection. Earlier consideration of extra-anatomic bypass may have been warranted when the pseudoaneurysm was identified. Arora et al reported that simple ligation of the femoral artery may be possible if Doppler signals from the dorsalis pedis artery are maintained.^6^ Although simple ligation may not be ideal in patients with a history of atherosclerotic occlusive disease, it could be considered as a life-saving option in patients without severe critical limb ischemia. In this patient, collateral circulation from the internal iliac artery to the deep femoral artery was well-developed, and simple ligation may not have worsened the ischemic symptoms. However, given the patient’s claudication and inability to walk more than 10 meters preoperatively, the decision to proceed with an extra-anatomic bypass was appropriate.

## Conclusions

This case illustrates the successful use of an extra-anatomic bypass to treat a groin infection. By avoiding the infected groin, infection control and vascular reconstruction were simultaneously achieved. Extra-anatomic bypass procedures are rare, and large-scale studies are difficult. The optimal approach should be determined on a case-by-case basis.

## Funding

None.

## Disclosures

None.

## References

[1] Engin C., Posacioglu H., Ayik F., Apaydin A.Z. (2005). Management of vascular infection in the groin. Tex Heart Inst J.

[2] Igari K., Kudo T., Katsui S., Nishizawa M., Uetake H. (2020). The comparison of long-term results between Aortofemoral and Axillofemoral bypass for patients with Aortoiliac occlusive disease. Ann Thorac Cardiovasc Surg.

[3] Altun G., Pulathan Z., Hemsinli D. (2018). Obturator bypass in the treatment of prosthetic graft infection: classic but still effective. Turk Gogus Kalp Damar Cerrahisi Derg.

[4] Lawson J.A. (1985). Surgical treatment of radiation induced atherosclerotic disease of the iliac and femoral arteries. J Cardiovasc Surg (Torino).

[5] Skowron K., Bauza-Kaszewska J., Kraszewska Z. (2021). Human skin microbiome: impact of intrinsic and extrinsic factors on skin microbiota. Microorganisms.

[6] Arora S., Weber M.A., Fox C.J., Neville R., Lidor A., Sidawy A.N. (2001). Common femoral artery ligation and local debridement: a safe treatment for infected femoral artery pseudoaneurysms. J Vasc Surg.

